# A Paraurethral Vaginal Mass in Rural Setting: A Case Report

**DOI:** 10.31729/jnma.6511

**Published:** 2021-06-30

**Authors:** Niresh Thapa, Subi Basnyat, Dilsahi Roka

**Affiliations:** 1 Department of General Practice and Emergency Medicine, Karnali Academy of Health Sciences, Jumla, Nepal; 2 Department of Obstetrics and Gynecology, Karnali Academy of Health Sciences, Jumla, Nepal

**Keywords:** *leiomyoma*, *rare diseases*, *rural health services*

## Abstract

Paraurethral vaginal leiomyoma is the infrequent case to be described. Approximately 300 cases have been described so far. Imaging modalities aid in identifying the morphological, structural characteristics of the mass and its relationship to the surrounding structures. Thirty-six years old married women presented with a vulvar mass of (3x5) cm^2^. Her associated complaints were left shift of the urinary stream and dyspareunia. Ultrasonography and cystography revealed a mass with no relationship with bladder or uterine structure. Surgical excision was performed. The histopathological report confirmed the diagnosis of paraurethral vaginal leiomyoma. Surgical excision is the treatment of choice and diagnosis is confirmed by histopathological examination.

## INTRODUCTION

A variety of pathologies from benign to malignant conditions like urethro-cystocele, Gartner's duct cyst, Skene duct cyst, urethral diverticulum, Bartholin gland cyst or vaginal malignancy should be considered in a vaginal wall mass.^1^ Vaginal and paraurethral leiomyoma are rare conditions to observe and difficult to diagnose at the time of presentation. Pre-operatively ultrasonography (USG) and magnetic resonance imaging (MRI) are indicated to establish morphology, location, and relationship of mass to the adjacent structures.^2^ Surgical excision is the treatment of choice.^3^ Histopathological examination (HPE) is the gold standard for diagnosis.^4^ We report a case of thirty-six years old married, women presented with vulvar mass.

## CASE REPORT

**Figure 1:**
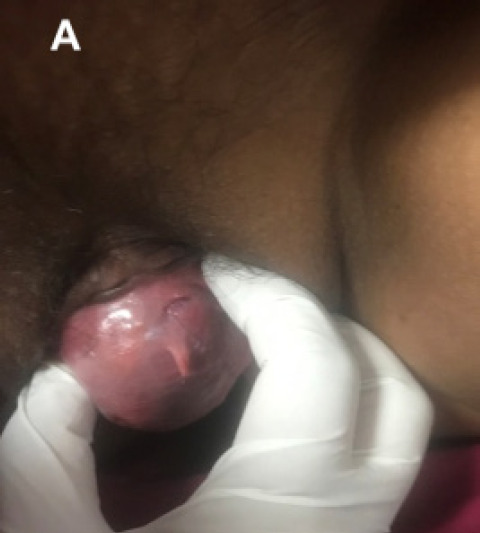
Preoperative (3x5)cm^2^ anterior vaginal mass.

Thirty-six years old married, parity three women presented with vulvar mass which had gradually increased in a year. She had a history of dyspareunia and a left shift of the urinary stream. There was no significant medical or surgical past history. Clinical examination revealed well defined, firm, solitary mass of (3x5) cm^2^. Vaginal epithelium over the mass had no rugosity and urethral meatus was shifted laterally to left (Figure 1).

USG revealed homogenous, hypoechoic mass with increased vascularity and no arising of the mass from any part of the bladder or uterine structure.

Cystography revealed no identification of the origin of mass from the bladder and urethral structure. The patient and her husband were counselled for surgical removal of mass with the provisional diagnosis of vaginal leiomyoma. Informed consent was obtained for the procedure.

**Figure 2:**
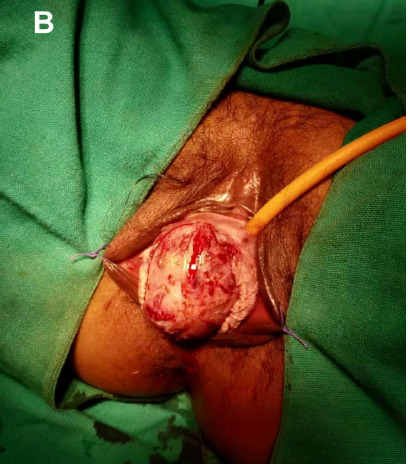
Intraoperative anterior vaginal mass.

A midline surgical incision was performed under spinal anaesthesia. A solid mass was encapsulated with no connection between the mass and the urethra (Figure 2).

**Figure 3:**
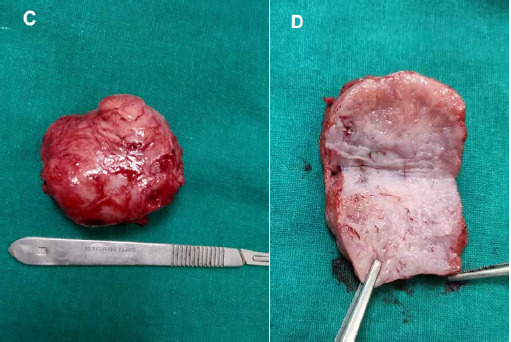
Enucleated and cut section of vaginal mass.

Sharp and blunt dissection was done to enucleate the mass (Figure 3). The wall was closed in two layers. Foley's catheter was inserted throughout the procedure. The patient was discharged in three days with no urinary complain. The patient was followed up after two weeks with a HPE report which was vaginal leiomyoma.

## DISCUSSION

Vaginal paraurethral leiomyoma is a rare benign mesenchymal tumor. Since its first description in 1733 by Denys de Leyden approximately 300 cases,^5^ have been reported with none from Nepal. The vaginal tumor is common in the reproductive age group between 35-50 years.^6^ The patient in our case was of age 36 years and ovarian hormone could stimulate the development of leiomyoma in this age group.^7^

Depending on the anatomic location and size of the mass the symptoms can vary from being asymptomatic to other symptoms like dyspareunia, dysuria, urinary tract infection, and obstructive voiding symptoms.^6,8^ The case presented to us with dyspareunia with no particular urinary complaints. Vagina leiomyoma indeed has been termed female prostate.^9^ Ischemia following a contraction of the smooth muscles could lead to pain,^10^ this could be the reason patient had tenderness during an examination.

There are four layers from anterior to posterior separating the exterior vaginal wall from the paraurethral space including epithelium, submucosa, fibromuscular band of smooth muscles, and periurethral fascia.^11^ Paraurethral leiomyoma occurs between the areas in the vesicovaginal septum or paraurethral space. A true paraurethral mass is when it has no connection to the urethra, bladder, or vagina.^12^ Initially in our case due to the anatomical location it was difficult to entitle as the vaginal or the paraurethral mass, following dissection it gave the impression of the paraurethral mass.

USG and MRI may help to estimate the extent, characterization, and involvement of the mass. Egbe, et al. suggested considering the assessment of lactate dehydrogenase (LDH) isoenzyme to differentiate the leiomyosarcoma.^2,13^ However, MRI and LDH assessment were limitations in our context.

Surgical excision is the treatment of choice. In 90% of cases, the vaginal route is favoured while in 10% of cases abdominal route is required.^3^ Detailed knowledge on the location of the mass, anatomy of the female urethra, and pelvic structure to facilitate reconstruction and familiarization with varieties of paraurethral, urethral, and vaginal mass is important before surgery. Although we performed surgery gonadotropin-releasing hormone analog and embolization can be used before surgery on the grounds of pathological diagnosis proven by biopsy.^14^

Paraurethtral vaginal leiomyoma is one of the rare kinds which is difficult to diagnose preoperatively. USG aids in identification in location, size of the mass, and its relationship to its adjacent structure.

Surgical excision via vaginal route is preferred but sound anatomical knowledge is important to facilitate a favourable patient outcome. Definite diagnosis is confirmed following the histopathological report.
